# Isolated septic arthritis of hip joint: a rare presentation of melioidosis. A case report

**DOI:** 10.1186/s13104-018-3171-6

**Published:** 2018-01-19

**Authors:** N. P. Weerasinghe, H. M. M. Herath, T. M. U. Liyanage

**Affiliations:** 1 Department of Microbiology, Faculty of Medicine, University of Ruhuna, P.O. Box 70, Galle, Sri Lanka; 2 Department of Medicine, Faculty of Medicine, University of Ruhuna, Galle, Sri Lanka; 3 University Unit, Teaching Hospital, Karapitiya, Galle, Sri Lanka

**Keywords:** Melioidosis, Isolated septic arthritis, Immune suppression, Sri Lanka

## Abstract

**Background:**

Despite, Sri Lanka lies in the melioidosis endemic belt between 5°N and 10°N surrounded by countries known to have endemic melioidosis for many years, comparatively fewer cases of melioidosis infection have been reported in Sri Lanka. Melioidosis has a wide spectrum of clinical presentation, ranging from severe pneumonia to abscess formation in various organs. Isolated septic arthritis, which is a rare but well-recognized manifestation of melioidosis, could be the sole presenting problem in some patients with melioidosis.

**Case presentation:**

We report a middle aged diabetic female who has been on azathioprine for autoimmune hepatitis, presenting with pain and swelling of left hip joint. Investigations confirmed the clinical suspicion of septic arthritis, but all relevant microbiological investigations failed to isolate a causative organism. Due to the history of diabetes, possible immunosuppression with azathioprine, and failure to recognise the possible causative organism by initial investigations prompted us to investigate for melioidosis. Diagnosis of melioidosis was made by presence high titre of antibodies to melioidin antigen, and rapid response to appropriate treatment. The patient was treated with intravenous imipenem 1000 mg 6 hourly and oral cotrimoxazole (1920 mg 12 hourly) for 4 weeks followed by eradication therapy with cotrimoxazole and doxycycline.

**Conclusion:**

Given that melioidosis-induced septic arthritis share common features with septic arthritis due to other common pyogenic bacteria, differentiation of these two conditions is extremely difficult. Therefore, melioidosis needs to be considered as a possibility, when a patient with risk factors for melioidosis such as diabetes or immunosuppression presents with isolated septic arthritis. This case report has been presented to raise the awareness of an unusual presentation of melioidosis; isolated septic arthritis.

## Background

Melioidosis is a pyogenic infection with high mortality and is caused by the facultative intracellular gram-negative bacterium; *Burkholderia pseudomallei* [1, 2]. It is endemic in tropical and subtropical zones of South East Asia and Northern Australia [2]. Despite, Sri Lanka lies in the melioidosis-endemic belt between 5°N and 10°N surrounded by countries known to have endemic melioidosis for many years, comparatively fewer cases of melioidosis infection have been reported in Sri Lanka [3]. Commonly identified risk factors for this infection are diabetes mellitus, heavy alcohol use, malignancy, chronic lung, liver and kidney disease and various other immune suppressive conditions [1]. *Burkholderia pseudomallei* enters the body through percutaneous inoculation or inhalation [1]. The disease is known as a remarkable imitator of other diseases such as tuberculosis, due to the wide and variable clinical spectrum of its manifestations [2]. As a result, a high index of clinical suspicion is required for the diagnosis. Delay in the diagnosis and failure to start appropriate and effective treatment against melioidosis, can worsen the outcome [4, 5].

When compared with other bacterial infections, melioidosis is a less common cause for isolated septic arthritis. Even though, melioidotic bone and joint infections are established entities, they are less commonly reported than other manifestations of melioidosis [6, 7]. Knee joint has been identified as the most commonly affected joint in melioidosis, followed by ankle, hip and shoulder joints [6]. Even though, melioidotic bone and joint infections have been reported in Sri Lanka previously [3, 8–10], isolated septic arthritis as the sole manifestation of melioidosis has not been reported in Sri Lanka up to now. Therefore, we report this unusual case of isolated septic arthritis of left hip joint due to melioidosis in a 45-year old female with multiple risk factors.

## Case presentation

A 45-year old Sinhalese woman from Galle district in Southern Province of Sri Lanka presented to our hospital with a 2 week history of intermittent fever and severely painful left hip for 3 days duration. She had a history autoimmune hepatitis for 6 months, for which she had been on azathioprine 50 mg daily, and poorly controlled type 2 diabetes mellitus for 11 years.

Fever was intermittent, initially low grade and later high grade, which abated with sweating, but not associated with chills and rigors. She also had mild sore throat and cough with constitutional symptoms. She was initially managed with oral co-amoxyclav as for possible upper respiratory tract infection by her family doctor. However, her fever continued and around 3 days prior to the admission, she has noticed severe pain in her left hip joint which has caused her to be increasingly bedbound and restricted left hip movement. Apart from these symptoms, she did not have involvement of other joints or haemoptysis. There was no previous history of tuberculosis or close contact with a patient with tuberculosis. She was a field officer, and had no exposure to soil; however, she occasionally had engaged in gardening.

**Fig. 1:**
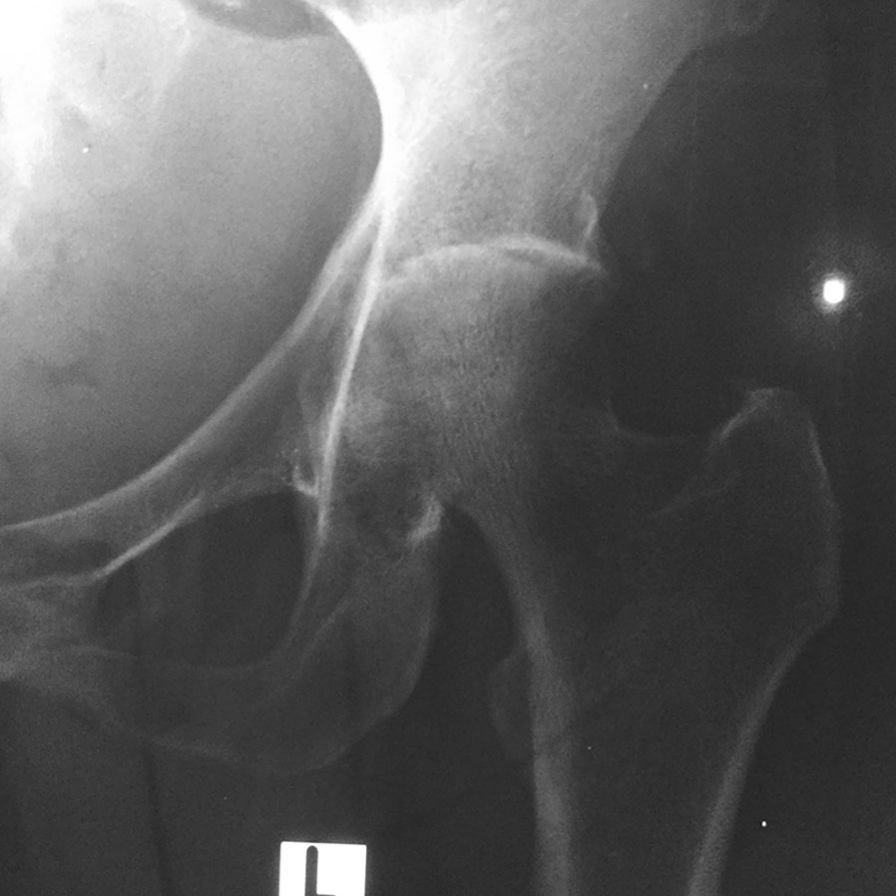
X-ray of the left hip showing narrow joint space and destruction of femoral head

**Fig. 2:**
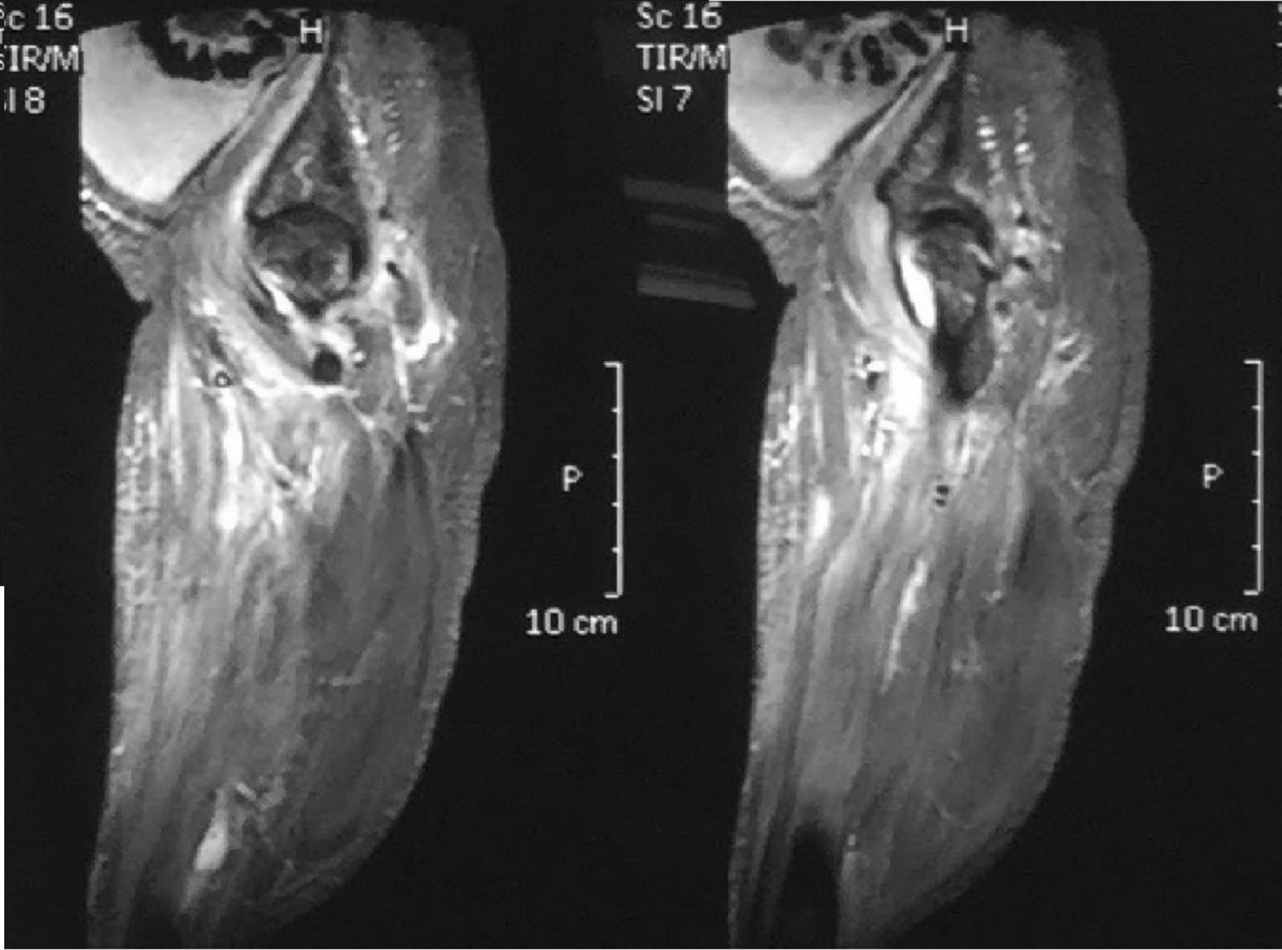
A coronal enhanced T1-weighted MRI image shows severe erosive bone changes, diffuse joint space narrowing and a small amount of joint effusion with synovial enhancement

On examination, she was ill looking and had a temperature of 40 °C. Examination revealed severely restricted movements of left hip. The joint was mildly warm to touch but there was no obvious swelling over it. Initial laboratory investigations revealed raised peripheral white cell count of 12.4 × 10^9^/L with 82% polymorphonuclear leukocytes, hemoglobin of 9.1 g/dL and a normal platelet count. Both erythrocyte sedimentation rate (65 mm/1st h) and C-reactive protein (86 mg/dL) were elevated. Initial radiograph of left hip joint was normal, but subsequent x-ray which was taken 1 week later showed a narrow joint space and destruction of femoral head (Fig. 1). Ultra sound scan of hip revealed small effusion with echogenic particles. MRI of hip was also suggestive of septic arthritis (Fig. 2).

After taking blood cultures, she was commenced on empirical antibiotic therapy with ceftriaxone IV 1 g 12 hourly and vancomycin IV 1 g 12 hourly. Despite antibiotic therapy, high fever spikes were continued and the clinical condition deteriorated. Repeated blood cultures and urine cultures revealed negative results. Joint fluid aspiration was failed since there was only a minimal amount of fluid. Further investigations including Mantoux test and sputum for acid fast bacilli (AFB) and mycobacterial culture were negative. Transthoracic 2D-echocardiogram did not show vegetations. Chest x-ray, ultrasound scan of abdomen and subsequently contrast CT scan of chest and abdomen did not reveal any significant abnormality such as visceral lymphadenopathy or abscesses. Autoimmune panel including ANA, ANCAs and serology for other less common infective causes like HIV, toxoplasmosis, and brucellosis were also negative.

Due to her immunosuppressive state and unresponsiveness to empirical antibiotic regimen, possibility of uncommon infective conditions was considered at this stage. One of the conditions that we considered was melioidosis since it is known to cause isolated septic arthritis in susceptible patients. Since the blood cultures were repeatedly negative, we performed antibodies to *B. pseudomallei*, which came positive with a titre of 1/10240 by Indirect Haemagglutination Assay (IHA). The presumptive diagnosis of melioidosis was made based on clinical and microbiological grounds. Definitive treatment was started with intravenous imipenem 1000 mg 6 hourly and oral co-trimoxazole (1920 mg 12 hourly). The patient responded well to the above treatment with fever settling within 72 h of starting treatment. While in the ward, she had intensive physiotherapy to improve mobilisation. At the end of 3 weeks, inflammatory markers came down to normal range and she was discharged on oral cotrimoxazole for another 10 weeks with a plan to have weekly reviews with full blood count and CRP.

## Discussion and conclusions

Melioidosis is caused by the gram-negative saprophyte *Burkholderia pseudomallei* [1] and it is mainly endemic to South East Asia and Northern Australia [2]; however, in recent years, there is increasing number of cases of melioidosis reported in Sri Lanka [3, 8, 11–14]. The spectrum of disease varies from mild self-limiting fever to severe septicemia with abscess formation in several organs [1]. It carries a high mortality unless treated early with appropriate antimicrobials.

Septic arthritis is a rare, but well recognized manifestation of melioidosis [6]. Clinically, it is difficult to differentiate from septic arthritis due to other common pathogens like *Staphylococcus aureus or Haemophilus influenzae*. Similar to other common pathogens, *Burkholderia pseudomallei* also affects large joints, such as knee, ankle, elbow, hip and shoulder [7]. However, unlike in our patient, knee joint is reported to be the most commonly involved joint in melioidosis [6]. However, a study conducted by Teparrakkul et al. [7] showed higher incidence of lower extremity joint involvement. In our patient, hip joint was the only affected joint. Even from Northern Australia and Thailand, where melioidosis is highly endemic, there is a paucity of evidence of such isolated involvement of hip joint. As far as we are aware, there have been no reported cases of isolated septic arthritis due to melioidosis, without other organ involvement from Sri Lanka. In one of the case series involving 540 patients, only four patients (< 1%) had septic arthritis [2]. Interestingly, in these case series conducted in different settings, blood cultures were reported to be positive in all patients with melioidotic septic arthritis [2, 15]. In contrast, blood cultures of our patient were repeatedly negative, and this could be due to prior antibiotic use. Even though, isolation of *B. pseudomallei* from the body fluids of patients remains the gold standard in the diagnosis, IHA has been used as the main serologic assay worldwide [16, 17]. Specificity of IHA is largely dependent on the prevalence of background sero-positivity. As it is high in endemic areas, IHA should be used cautiously in the diagnosis of melioidosis in endemic regions [16]. However, it may have a place in the diagnosis of active infection in non-endemic areas [17, 18]. Even though, Sri Lanka is now considered as endemic area with increasing number of cases reported in last decade, IHA may still be useful in selected patients like ours if the clinical suspicion is high. Diagnosis of melioidotic septic arthritis is made in our patient based on the presence of very high titre of antibody (IHA), good response to specific treatment targeting melioidosis and exclusion of other possible causative organisms.

One of the most important risk factor for melioidotic septic arthritis is diabetes mellitus; however, underlying immunosuppression is also an important risk factor [6, 15]. Our patient had both these risk factors. In melioidotic joint infections, the presenting symptom is usually persistent fever rather than shock or respiratory failure, and overall mortality is low among patients with melioidotic joint infections [7]. Our patient too had persistent fever with no evidence of haemodynamic compromise or respiratory distress. Even with significant damage to the hip joint, our patient had a remarkable recovery and was able to walk with minimal assistance at the end of 3 weeks of treatment.

In conclusion, isolated septic arthritis, a rare but a recognized manifestation of melioidosis, could be the presenting problem in some patients with melioidosis. This interesting case highlights the importance of considering “melioidosis” in susceptible patients presenting with isolated septic arthritis, even if the other manifestations of melioidosis (abscess, lung involvement) are not present. Our case report also illustrates the difficulty in isolating *B. pseudomallei* especially when there is prior antibiotic use. This case further illustrates the fact that the IHA, even though not very specific test particularly in endemic areas, can be used cautiously to diagnose melioidosis.

## Abbreviations

AFB: acid fast bacilli
2D-echocardiogram: two-dimensional echocardiography
CT: computed tomography
ANA: antinuclear antibody
ANCAs: anti-neutrophil cytoplasmic antibodies
HIV: human immunodeficiency virus
